# Supplementary material for: Investigation of Metastable Low Dimensional Halometallates
Source: Molecules. 2022 Jan 3;27(1):280. doi: 10.3390/molecules27010280 (PMC8746344; doi:10.3390/molecules27010280)
Supplement: Supplementary file 1 [file molecules-27-00280-s001.zip › molecules-1500489-supplementary.pdf]

Supplementary Data

for

Investigation of Metastable Low Dimensional Halometallates

Navindra Keerthisinghe, Matthew S. Christian†, Anna A. Berseneva, Gregory Morrison, Mark D. Smith, Vladislav V. Klepov‡ and Hans-Conrad zur Loye*

Department of Chemistry and Biochemistry, University of South Carolina, Columbia, SC, 29208, USA

*Correspondence: zurloye@mailbox.sc.edu

†currently at: Department of Geochemistry, Sandia National Laboratories, Albuquerque, NM, 87123, USA

‡currently at: Department of Chemistry, Northwestern University, Evanston, IL, 60208, USA

Table of Contents

1. Figures of Unit Cells *3*
2. Supplementary Crystal Structure Figures *6*
3. Powder X-ray Diffraction Data *7*
4. Infrared spectra calculations *11*
5. Bandgap calculations *14*
6. Energy Dispersive Spectroscopy (EDS) and TGA data *14*
7. Crystal Morphology *18*
8. Selected Interatomic Distances and Bond Angles for 1–5 .... *18*
9. References *18*

1. Figures of Unit Cells

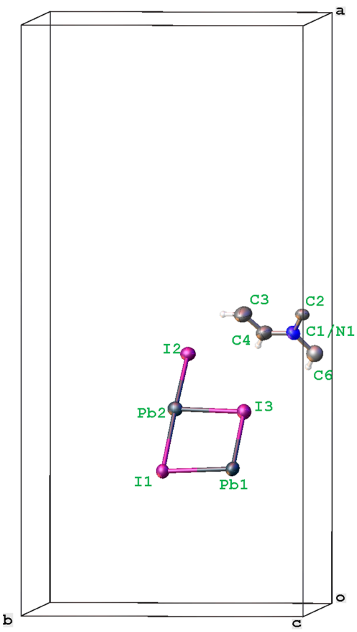

Figure S1. Asymmetric unit of [1,10-phenH][$Pb_{3.5}I_8$]. Pb, I, C, N and H atoms are shown by dark gray, purple, light gray, blue, and white spheres respectively. Note that only quarter of the 1,10-phenanthroline molecule is visible due to high symmetry.

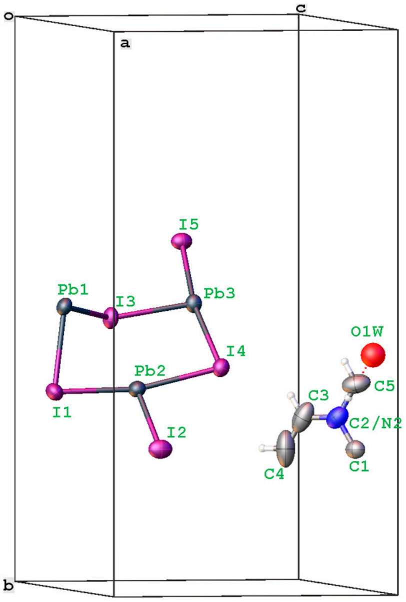

Figure S2. Asymmetric unit of [1,10-phenH_2][Pb_5I_{12}]·(H_2O). Pb, I, C, N, O and H atoms are shown by dark gray, purple, light gray, blue, red, and white spheres respectively.

Note that only quarter of the 1,10-phenanthroline molecule is visible due to high symmetry.

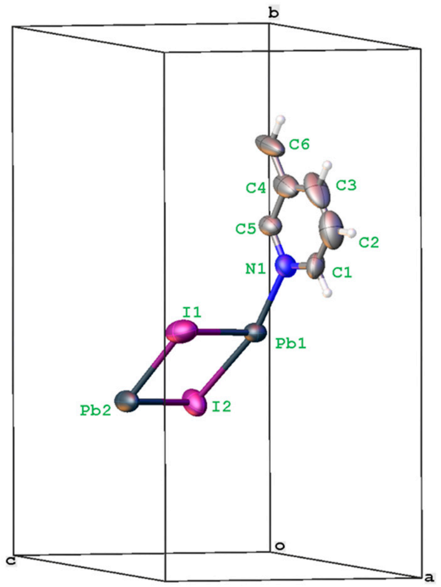

Figure S3. Asymmetric unit of [1,10-phen][Pb_2I_4]. Pb, I, C, N, and H atoms are shown by dark gray, purple, light gray, blue, and white spheres respectively. Note that only half of the 1,10-phenanthroline molecule is visible due to high symmetry.

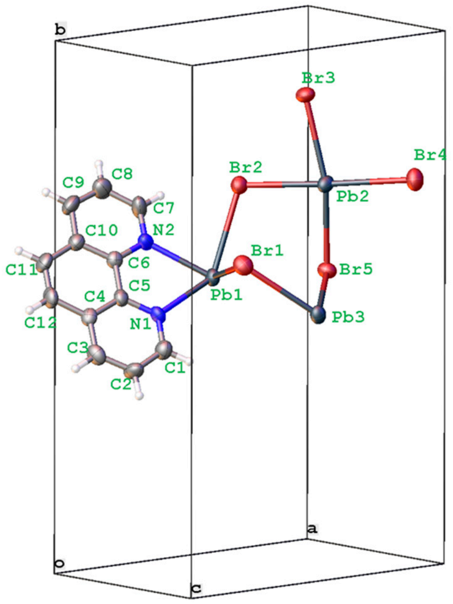

Figure S4. Asymmetric unit of [1,10-phen]$_2$[Pb_5Br_{10}]. Pb, Br, C, N and H atoms are shown by dark gray, red, light gray, blue, and white spheres respectively.

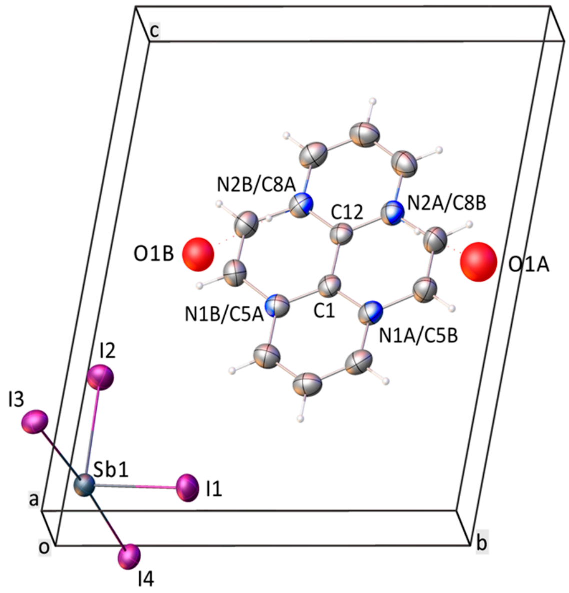

Figure S5. Asymmetric unit of [1,10-phenH][SbI_4]·(H_2O). Sb, I, C, N, O and H atoms are shown by dark gray, purple, light gray, blue, red and white spheres respectively.

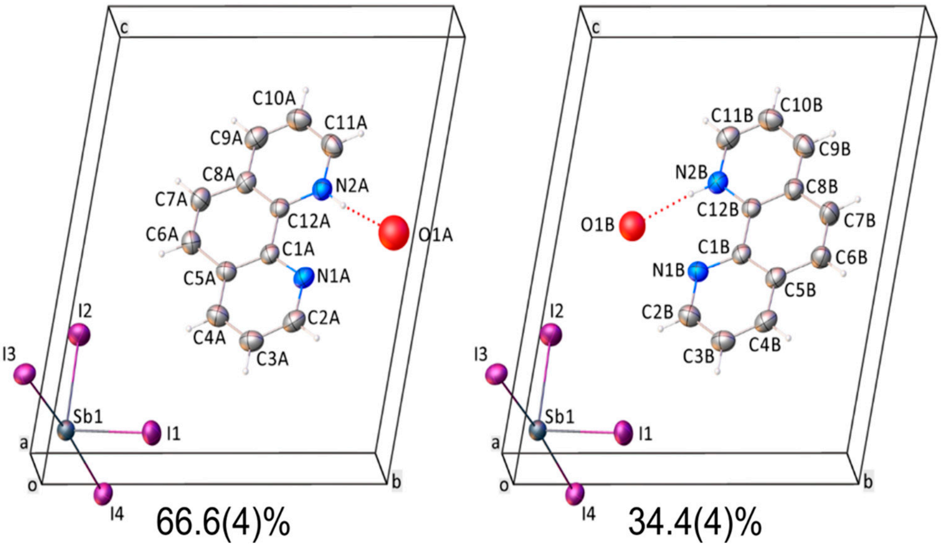

Figure S6. The two [1,10-phenH]$^+$/H_2O disorder components, with occupancies. Sb, I, C, N, O and H atoms are shown by dark gray, purple, light gray, blue, red and white spheres respectively.

2. Supplementary Crystal Structure Figures

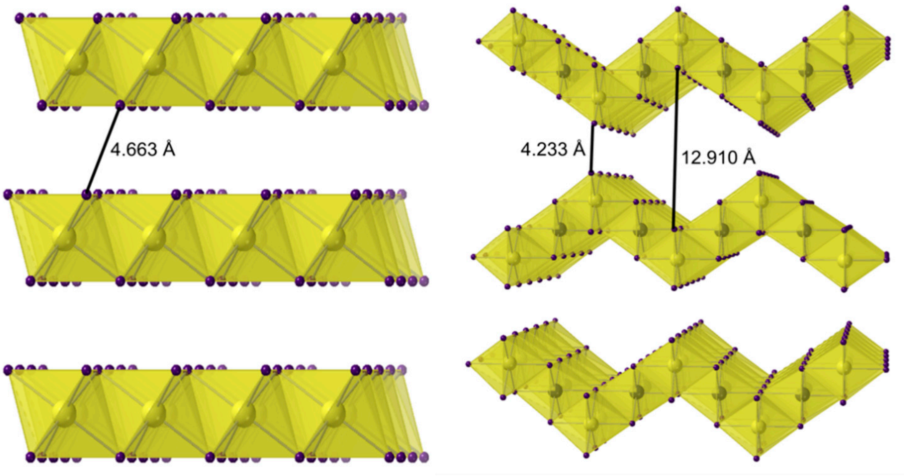

Figure S7. Stacking arrangement of layers of PbI_2 (left) and [1,10-phenH][$Pb_{3.5}I_8$] (right). The disordered [1,10-phenH]$^+$ cations are not shown for clarity. Pb and I atoms are shown by yellow and purple spheres.

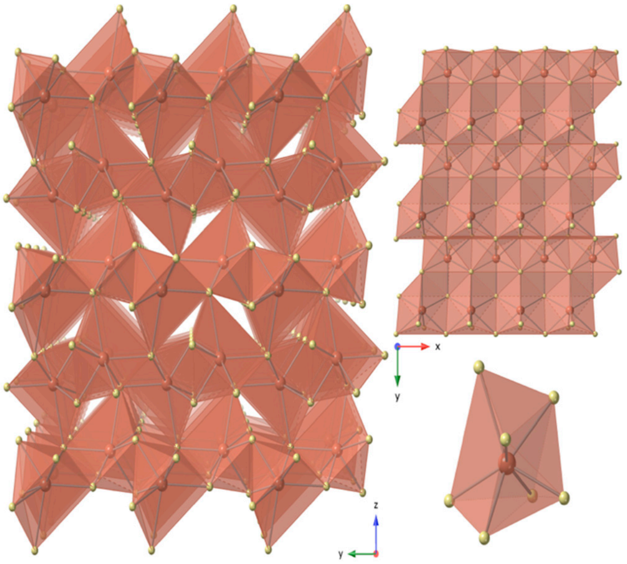

Figure S8. Crystal structure of $PbBr_2$ view down a-axis (left). View of single layer in $PbBr_2$ down *c*-axis (top right) and $PbBr_7$ unit (bottom right). Pb in beige color polyhedra, Br in yellow spheres.

3. Powder X-ray Diffraction Data

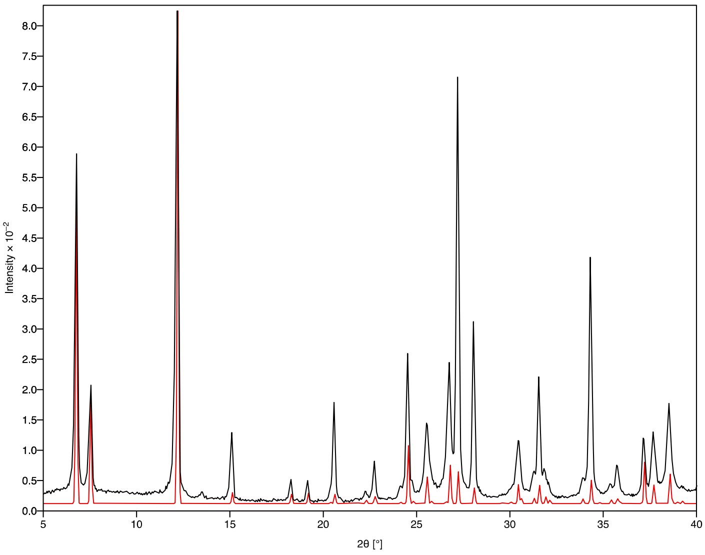

Figure S9. PXRD pattern for [1,10-phenH][$Pb_{3.5}I_8$]. Peak intensity mismatch due to preferred orientations along (310). Experimental pattern and calculated patterns are shown in black and red lines.

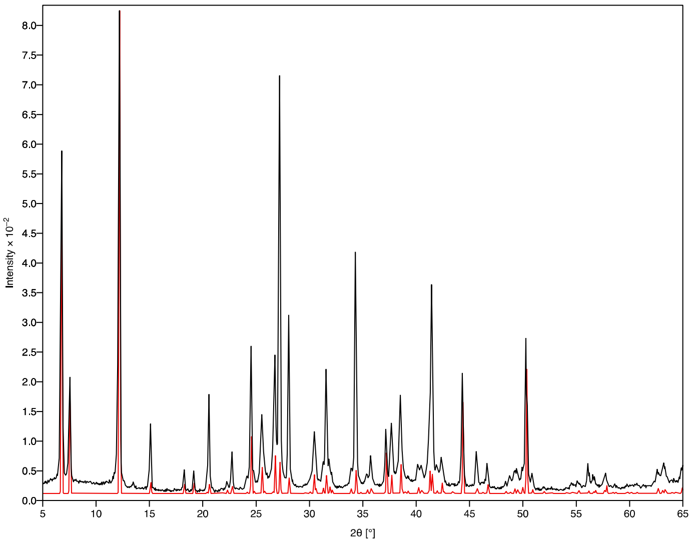

Figure S10. Long range PXRD pattern for [1,10-phenH][$Pb_{3.5}I_8$]. Peak intensity mismatch due to preferred orientations along (310). Experimental pattern and calculated patterns are shown in black and red lines.

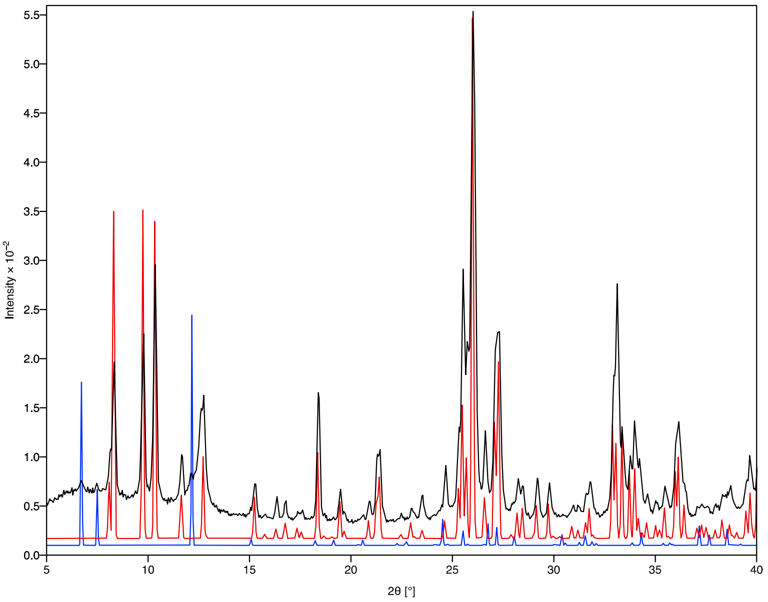

Figure S11. PXRD pattern for [1,10-phenH_2][Pb_5I_{12}]·(H_2O). Crystals exhibit preferred orientations along (440) and minor peaks of material **1**. The sample was measured on a zero-background slide due to low yield. Experimental pattern in black, and calculated patterns for [1,10-phenH_2][Pb_5I_{12}]·(H_2O) and [1,10-phenH][$Pb_{3.5}I_8$] are shown in red and blue lines respectively.

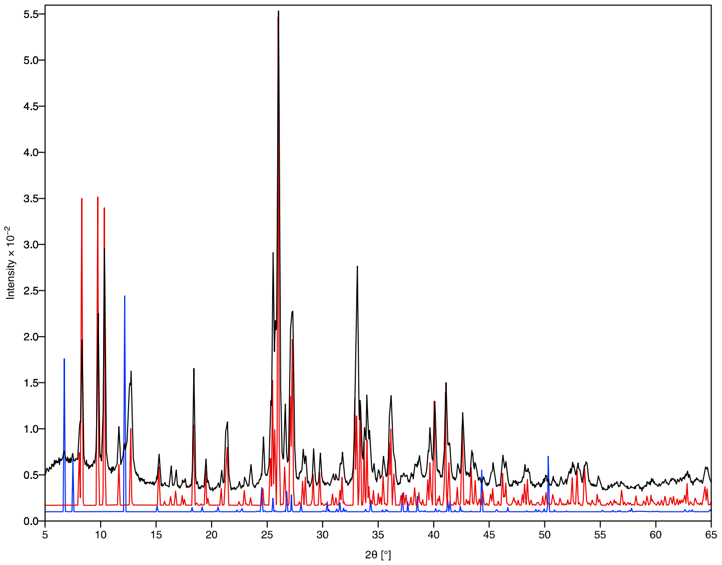

Figure S12. Long range PXRD pattern for [1,10-phenH_2][Pb_5I_{12}]·(H_2O). Crystals exhibit preferred orientations along (440) and minor peaks of material **1**. The sample was measured on a zero-background slide due to low yield. Experimental pattern in

black, and calculated patterns for [1,10-phenH$_2$][Pb$_5$I$_{12}$]·(H$_2$O) and [1,10-phenH][Pb$_{3.5}$I$_8$] are shown in red and blue lines respectively.

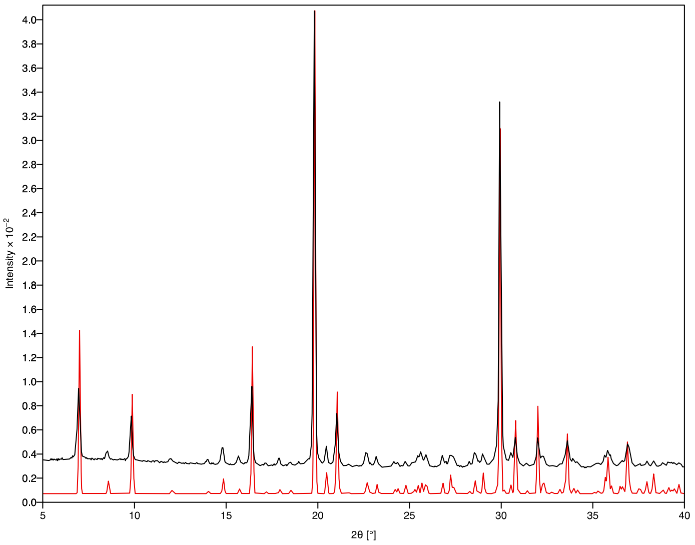

Figure S13. PXRD pattern for [1,10-phen]$_2$[Pb$_5$Br$_{10}$]. Crystals exhibit preferred orientations along (040). The sample was measured on a zero-background slide due to low yield. Experimental pattern and calculated patterns are shown in black and red lines.

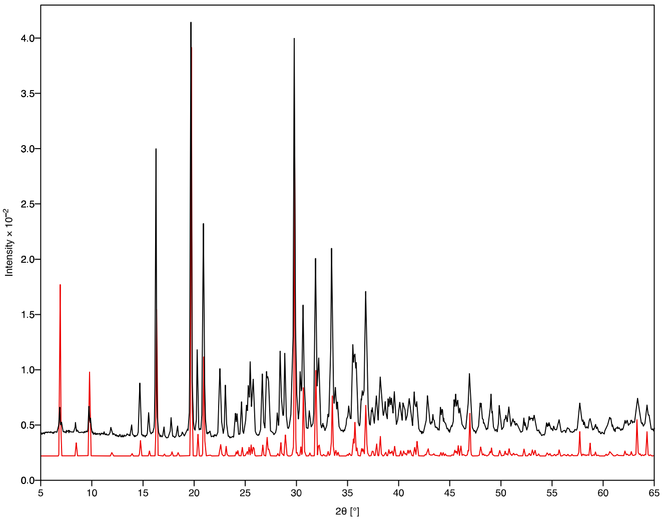

Figure S14. Long rage PXRD pattern for [1,10-phen]$_2$[Pb$_5$Br$_{10}$]. Crystals exhibit preferred orientations along (040). The sample was measured on a zero-background slide due to low yield. Experimental pattern and calculated patterns are shown in black and red lines.

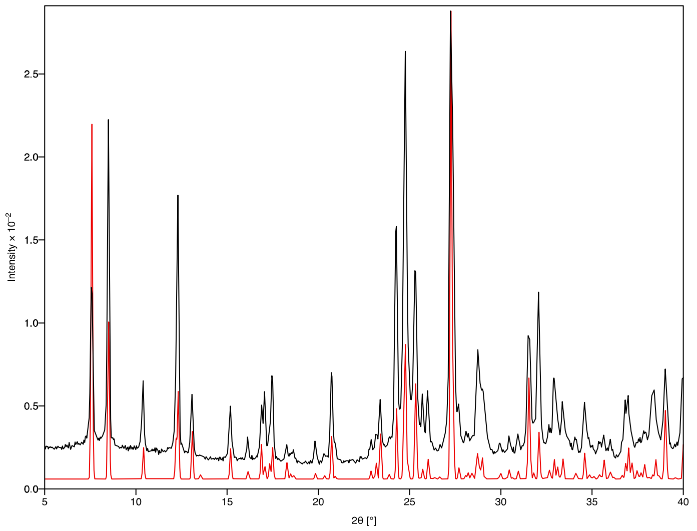

Figure S15. PXRD pattern for [1,10-phenH][SbI_4]·(H_2O). Peak intensity mismatch due to preferred orientations along (202). Experimental pattern and calculated patterns are shown in black and red lines.

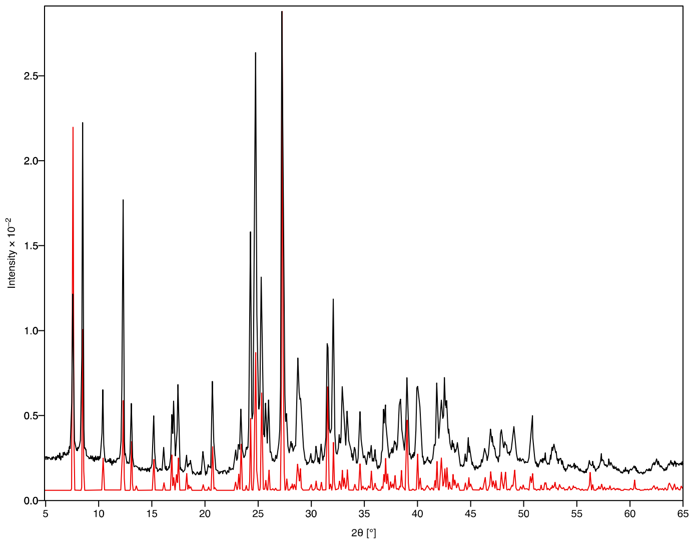

Figure S16. Full range PXRD pattern for [1,10-phenH][SbI_4]·(H_2O). Peak intensity mismatch due to preferred orientations along (202). Experimental pattern and calculated patterns are shown in black and red lines.

4. Infrared spectra calculations

To develop an understanding of shifts in IR active N–H stretch, ν(N–H), between $[1,10\text{-phenH}]^{+}$ and $[1,10\text{-phenH}_2]^{2+}$ species and hence distinguish those species in experimental FTIR-spectra, we attempted a series of vibrational frequency calculations. Ground state geometry optimization and vibrational frequency calculations for phen molecule and $[1,10\text{-phenH}]^{+}$ and $[1,10\text{-phenH}_2]^{2+}$ species was performed using density functional calculation (DFT) with the B3LYP-D3 functional paired with the 6-31+G* basis. The method for calculation of IR band frequencies was selected after additional calculations, including ground-state geometry optimization at the B3LYP, B3LYP-D3, LRC- ωPBEh. wB97X level using the 6-31+G* and 6-311+G** bases, which yielded marginal differences in frequencies. The B3LYP-D3 functional was chosen, as it demonstrated the best agreement with the experimental spectra of 1,10-phenanthroline. All calculations were performed using Spartan18 software [51].

In relation to current experiment, we studied 4000–2000 cm^{-1} range in IR spectra and assigned IR bands to specific bond stretch. Calculated IR spectrum for $[1,10\text{-phenH}]^{+}$ ion contains the peak at 3458 cm^{-1} corresponding to N–H stretch (Figure S17) while $[1,10\text{-phenH}_2]^{2+}$ cation has two N–H stretches: weak asymmetric (3561 cm^{-1}) and strong symmetric (3578 cm^{-1}). N–H stretch in $[1,10\text{-phenH}]^{+}$ is red-shifted on 120 cm^{-1} compare to N–H stretch in $[1,10\text{-phenH}_2]^{2+}$ suggesting that N–H bond in $[1,10\text{-phenH}_2]^{2+}$ is stronger than in $[1,10\text{-phenH}]^{+}$. Indeed, N–H bonds in the optimized structures are equal to 1.015 Å and 1.024 Å for $[1,10\text{-phenH}_2]^{2+}$ and $[1,10\text{-phenH}]^{+}$, respectively. Such a trend in N–H bonding is a result of a lone pair in $[1,10\text{-phenH}]^{+}$ that attracts a proton from the NH^{+} moiety and hence elongates N–H bond and shifts N–H stretch to smaller energies. Calculated IR spectra trend is in line with experiment where strong broad N–H stretch for $[1,10\text{-phenH}]^{+}$ at ≈2700 cm^{-1} shifted on ≈150 cm^{-1} to ≈2850 cm^{-1} for $[1,10\text{-phenH}_2]^{2+}$. At the same time, peak position for N–H stretch in theoretical and experimental IR spectra

differ on ≈750 cm^{-1}. In the presented calculations geometry optimization and vibrational frequency were calculated in the gas phase [52], however, due to hydrogen bonding or coulombic interaction of NH^+ moiety in the solid state, N–H stretch in pyridinium-based salts appears in 3300–2375 cm^{-1} region [33]. For 1,10-phenanthroline molecule which does not contain protonated pyridine group, there is only C–H stretches in the range 4000–2000 cm^{-1}. Corresponding calculated IR C–H stretches located at 3210–3160 cm^{-1} and 3250–3200 cm^{-1} for 1,10-phenanthroline molecule and protonated $[1,10\text{-phenH}]^+$ and $[1,10\text{-phenH}_2]^{2+}$, respectively (Figure S17). From the experimental IR-spectra for 1,10-phenanthroline, $[1,10\text{-phen}]_2[Pb_5Br_{10}]$, and $[1,10\text{-phen}][Pb_2I_4]$ we observed several medium IR-bands at ≈3050 cm^{-1} that can be assigned to C–H stretches of 1,10-phenanthroline and that are hidden for other compositions containing $[1,10\text{-phenH}_2]^{2+}$ and $[1,10\text{-phenH}]^+$ due to broad N–H stretch. Therefore, vibrational frequencies calculations in the gas phase allowed to explain the shift in IR for N–H stretch relevant to protonation number and hence corroborated the composition of the studied compounds.

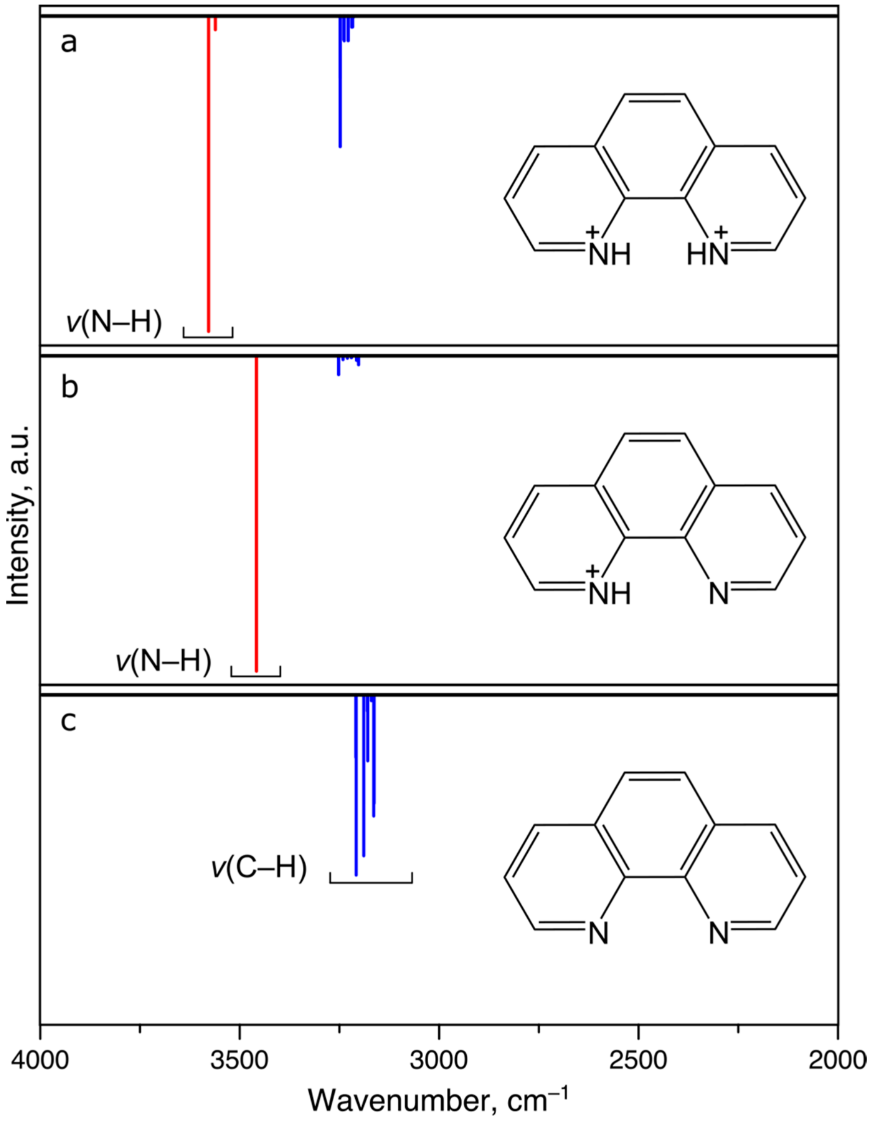

Figure S17. Calculated IR spectra for (*a*) 1,10-phenantrolin-1,10-diium cation, [1,10-phenH_2]$^{2+}$, (*b*) 1,10-phenanthrolin-1-ium ion, [1,10-phenH]$^+$, and (*c*) 1,10-phenanthroline molecule, in the range 4000–2000 cm^{-1}. Red and blue lines highlight N–H and C–H bonds' stretches, respectively.

5. Bandgap calculations

Bandgap calculations were carried out for [1,10-phenH][$Pb_{3.5}I_8$] in order to gain a better understanding of the material's electronic structure. The calculated band structure for one orientation of the disordered phenanthroline is shown in Figure S18. Our calculations failed to predict a band gap even though diffuse reflectance spectra show the compound has a band gap of 2.87 eV. The failure to predict a band gap is due to several known limitations of semi-local DFT functionals. Previous calculations have been able to obtain good band gap predictions with semi-local functionals [53-57]. However, these calculations did not include a Van der Waals correction for crystal structure optimization. This results in longer than experiment bond lengths. In these same studies, the HSE or PBE0 hybrid-functional predicts larger band-gap values than experiment. Therefore, using the HSE or PBE0 functional in combination with the XDM dispersion correction likely would have yielded a predicted band gap in this material while accurately modeling the structure energetics. This approach was not used because of the greater computational resources required to use the HSE functional.

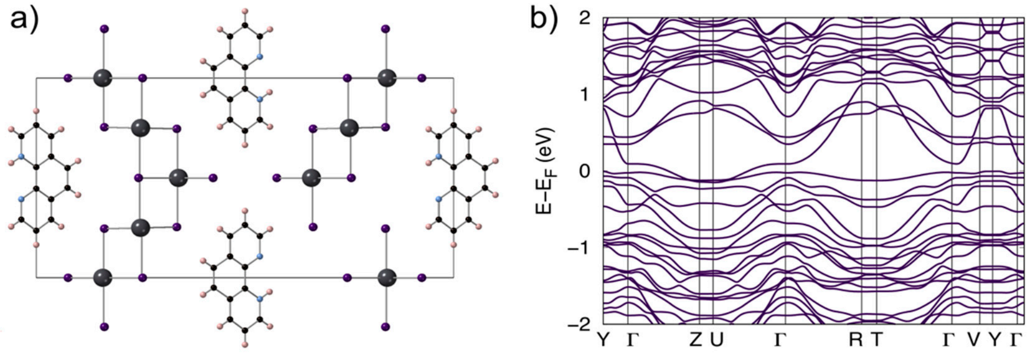

Figure S18. Model unit cell of [1,10-phenH][$Pb_{3.5}I_8$] (a) and calculated band structure (b).

6. Energy Dispersive Spectroscopy (EDS) and TGA data.

EDS was performed directly on crystals mounted on an SEM stub with carbon tape. Elemental analysis was done using a Tescan Vega-3 SEM instrument equipped with

a Thermo EDS attachment. The SEM was operated in low-vacuum mode with a 20 kV accelerating voltage and a 20 s accumulating time.

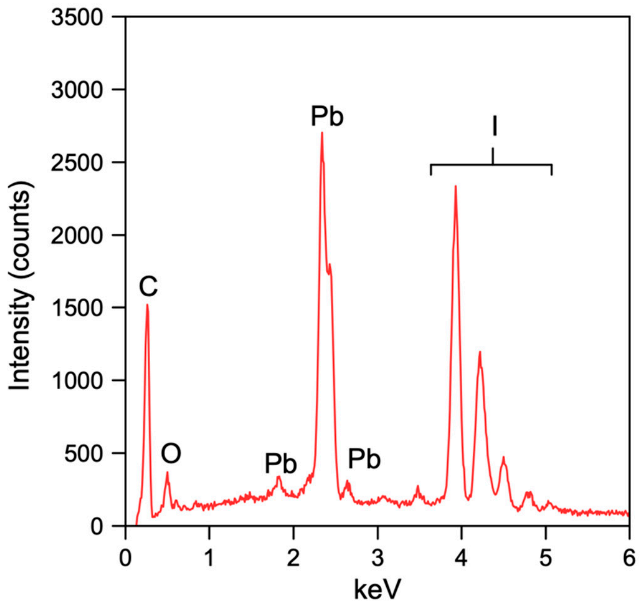

Figure S19. EDS spectrum for [1,10-phenH][$Pb_{3.5}I_8$].

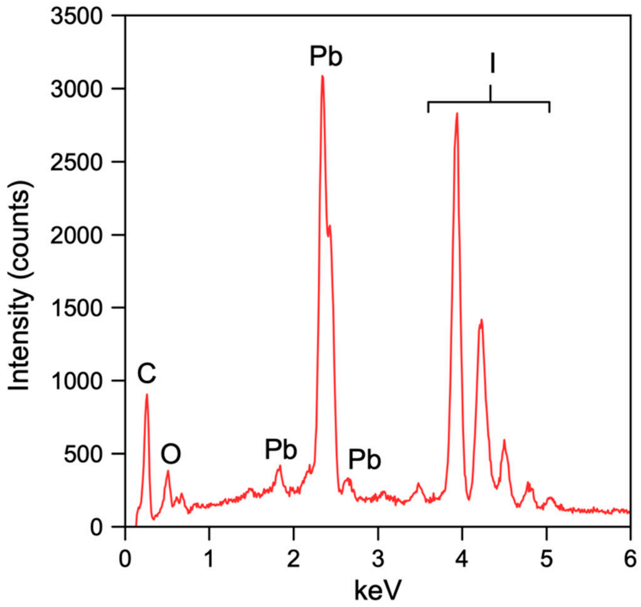

Figure S20. EDS spectrum for [1,10-phenH_2][Pb_5I_{12}]·(H_2O).

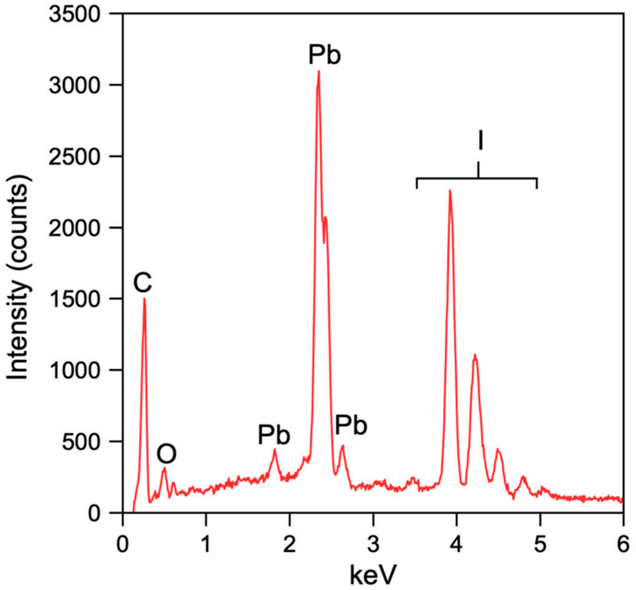

Figure S21. EDS spectrum for [1,10-phen][Pb_2I_4].

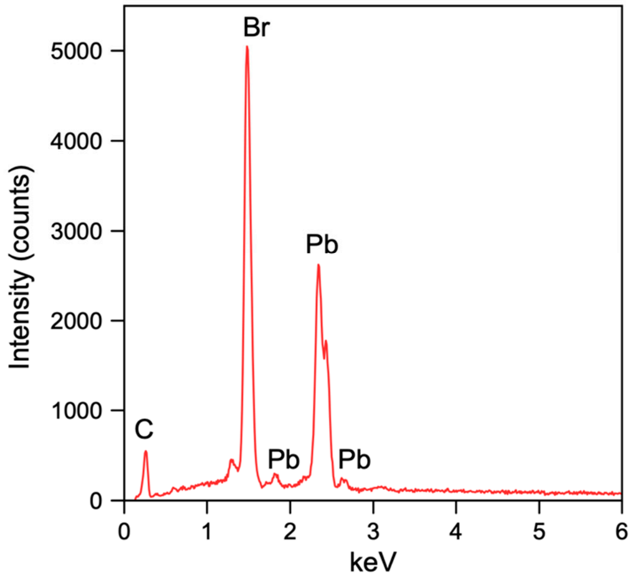

Figure S22. EDS spectrum for $[1,10\text{-phen}]_2[Pb_5Br_{10}]$.

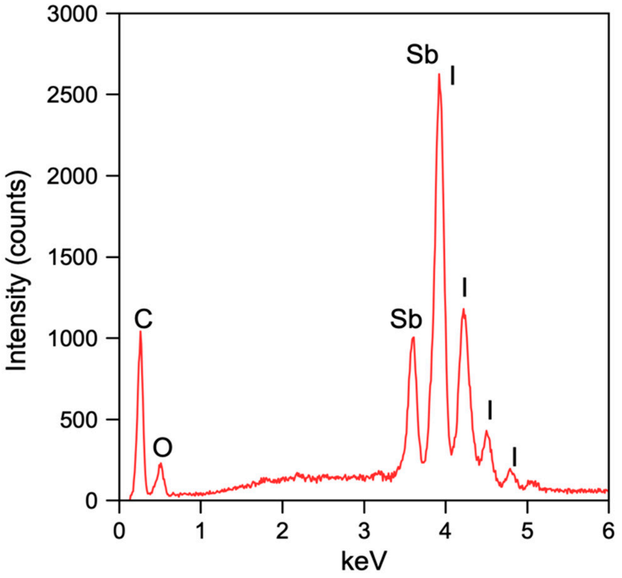

Figure S23. EDS spectrum for [1,10-phenH][SbI_4]·(H_2O).

C H N analysis was performed on (PhenH)SbI_4•H_2O, as we could be certain that the sample could be collected phase pure. The other materials co-crystallized, making the isolation of a guaranteed phase pure sample questionable. The results for (PhenH)SbI_4•H_2O came out as C 17.48 (17.39 calc), H 1.32 (1.34 Calc), N 3.35 (3.38 Calc).

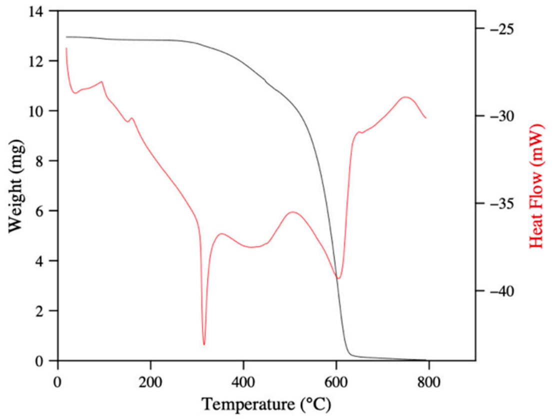

Figure S24. TGA plot for (phenH)$Pb_{3.5}I_8$.

7. Crystal Morphology

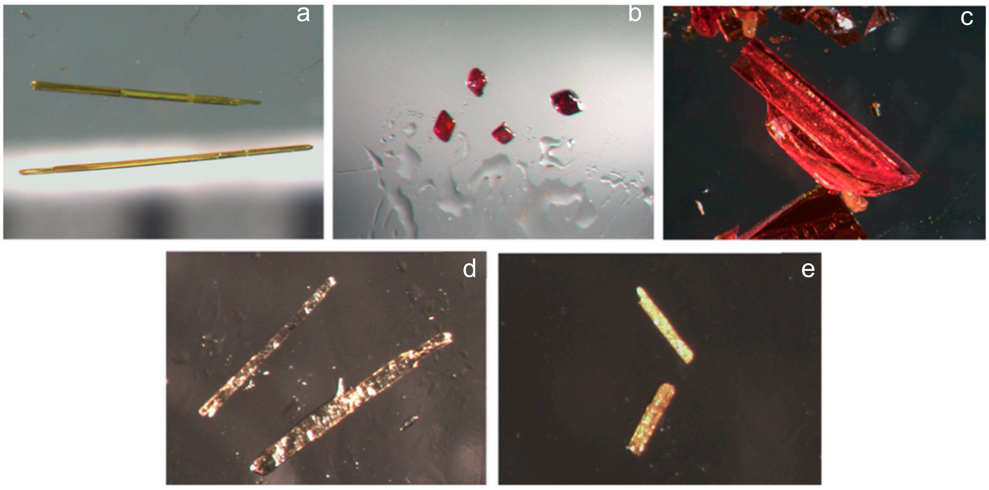

Figure S25. Single crystals of a) [1,10-phenH][$Pb_{3.5}I_8$], b) red diamond like crystals obtained from evaporated samples, c) single crystals of [1,10-phenH_2][Pb_5I_{12}]·(H_2O), d) single crystals of [1,10-phen][Pb_2I_4] and e) single crystals of [1,10-phenH][SbI_4]·(H_2O).

8. Selected Interatomic Distances and Bond Angles for **1**–**5**.

Table S1. Selected Interatomic Distances (Å) for [1,10-phenH][$Pb_{3.5}I_8$].

Atom	Atom	Length/Å	Atom	Atom	Length/Å
Pb1	I1	3.2744(5)	Pb2	I3	3.2223(9)
Pb1	I1	3.2744(5)	Pb2	I3	3.2223(9)
Pb1	I3	3.1928(10)	C1	C2	1.397(15)
Pb1	I3	3.1779(10)	C1	C4	1.33(2)
Pb1	I3	3.1928(10)	C1	C6	1.46(3)
Pb1	I3	3.1779(10)	C2	C2	1.41(3)
Pb2	I1	3.3432(15)	C3	C4	1.40(2)
Pb2	I1	3.3580(14)	C3	C4	1.40(2)
Pb2	I2	3.1246(15)	C6	C6	1.31(6)
Pb2	I2	3.1100(15)			

Table S2. Selected Bond Angles for [1,10-phenH][$Pb_{3.5}I_8$].

Atom	Atom	Atom	Angle/°	Atom	Atom	Atom	Angle/°
I1	Pb1	I1	180.0	I3	Pb2	I1	87.52(2)
I3	Pb1	I1	89.74(3)	I3	Pb2	I1	89.77(2)
I3	Pb1	I1	90.27(3)	I3	Pb2	I1	87.52(2)
I3	Pb1	I1	89.73(3)	I3	Pb2	I1	89.77(2)
I3	Pb1	I1	90.26(3)	I3	Pb2	I3	175.05(4)
I3	Pb1	I1	91.53(3)	Pb1	I1	Pb1	174.90(4)
I3	Pb1	I1	88.47(3)	Pb1	I1	Pb2	89.23(2)
I3	Pb1	I1	91.53(3)	Pb1	I1	Pb2	87.51(2)
I3	Pb1	I1	88.47(3)	Pb1	I1	Pb2	87.51(2)
I3	Pb1	I3	90.52(2)	Pb1	I1	Pb2	89.23(2)
I3	Pb1	I3	89.48(2)	Pb2	I1	Pb2	84.00(3)
I3	Pb1	I3	180.0	Pb2	I1	Pb2	91.98(4)
I3	Pb1	I3	89.48(2)	Pb1	I3	Pb1	89.48(2)
I3	Pb1	I3	180.0	Pb1	I3	Pb2	91.03(3)
I3	Pb1	I3	90.52(2)	Pb1	I3	Pb2	93.41(3)
I1	Pb2	I1	84.00(3)	C2	C1	C6	119.6(16)
I2	Pb2	I1	92.02(4)	C4	C1	C2	119.2(15)
I2	Pb2	I1	176.02(4)	C4	C1	C6	121.1(18)
I2	Pb2	I1	176.00(4)	C1	C2	C1	120.7(16)
I2	Pb2	I1	91.99(4)	C1	C2	C2	119.6(8)
I2	Pb2	I2	91.98(4)	C1	C2	C2	119.6(8)
I2	Pb2	I3	92.47(2)	C4	C3	C4	118.0(2)
I2	Pb2	I3	92.47(2)	C1	C4	C3	121.5(19)
I2	Pb2	I3	90.05(2)	C6	C6	C1	120.6(13)
I2	Pb2	I3	90.05(2)				

Table S3. Selected Interatomic Distances (Å) for [1,10-phenH_2][Pb_5I_{12}]·(H_2O) .

Atom	Atom	Length/Å	Atom	Atom	Length/Å
Pb3	I4	3.3100(3)	Pb2	I4	3.2860(5)
Pb3	I4	3.3101(3)	Pb2	I1	3.1400(5)
Pb3	I5	3.1352(3)	Pb2	I2	3.0990(4)
Pb3	I5	3.1352(3)	Pb2	I2	3.0989(4)
Pb3	I3	3.2111(3)	C1	C1	1.425(13)
Pb3	I3	3.2111(3)	C1	N2	1.377(6)
Pb1	I3	3.2057(3)	C1	N2	1.377(6)
Pb1	I3	3.2057(3)	N2	C3	1.369(8)
Pb1	I3	3.2058(3)	N2	C5	1.471(13)
Pb1	I3	3.2057(3)	C4	C3	1.358(9)
Pb1	I1	3.1945(4)	C4	C3	1.358(9)
Pb1	I1	3.1945(4)	C5	C5	1.34(3)

Table S4. Selected Bond Angles for [1,10-phenH_2][Pb_5I_{12}]·(H_2O).

Atom	Atom	Atom	Angle/°	Atom	Atom	Atom	Angle/°
I4	Pb3	I4	92.023(12)	I1	Pb1	I3	94.436(8)
I5	Pb3	I4	89.111(7)	I1	Pb1	I3	85.565(8)
I5	Pb3	I4	89.112(7)	I1	Pb1	I3	85.564(8)
I5	Pb3	I4	170.311(7)	I1	Pb1	I3	94.436(8)
I5	Pb3	I4	170.310(7)	I1	Pb1	I1	180.0
I5	Pb3	I5	91.388(12)	I1	Pb2	I4	167.836(14)
I5	Pb3	I3	84.089(7)	I2	Pb2	I4	91.689(13)
I5	Pb3	I3	84.089(7)	I2	Pb2	I4	91.688(13)
I5	Pb3	I3	97.260(7)	I2	Pb2	I1	96.959(13)
I5	Pb3	I3	97.262(7)	I2	Pb2	I1	96.959(13)
I3	Pb3	I4	86.249(10)	I2	Pb2	I2	89.162(13)
I3	Pb3	I4	92.417(10)	Pb3	I4	Pb3	87.977(12)
I3	Pb3	I4	86.249(10)	Pb2	I4	Pb3	90.098(9)
I3	Pb3	I4	92.415(10)	Pb2	I4	Pb3	90.098(9)
I3	Pb3	I3	178.080(14)	Pb3	I5	Pb3	88.613(12)
I3	Pb1	I3	88.907(11)	Pb1	I3	Pb3	132.257(11)
I3	Pb1	I3	88.906(11)	Pb2	I1	Pb1	81.740(11)
I3	Pb1	I3	180.0	Pb2	I2	Pb2	88.613(13)
I3	Pb1	I3	91.093(11)	N2	C1	N2	117.8(7)
I3	Pb1	I3	180.0	C1	N2	C5	118.1(7)
I3	Pb1	I3	91.094(11)	C3	N2	C1	120.7(6)
I1	Pb1	I3	85.564(8)	C3	N2	C5	121.3(7)
I1	Pb1	I3	94.435(8)	C3	C4	C3	118.7(10)
I1	Pb1	I3	94.436(8)	C4	C3	N2	121.1(8)
I1	Pb1	I3	85.564(8)	C5	C5	N2	120.8(5)

Table S5. Selected Interatomic Distances (Å) for [1,10-phen][Pb_2I_4].

Atom	Atom	Length/Å	Atom	Atom	Length/Å
Pb1	I2	3.3167(9)	Pb2	I1	3.1305(12)
Pb1	I2	3.3168(9)	N1	C5	1.367(14)
Pb1	I1	3.1990(10)	N1	C1	1.297(13)
Pb1	I1	3.1991(10)	C5	C5	1.42(2)
Pb1	N1	2.484(8)	C5	C4	1.416(15)
Pb1	N1	2.484(8)	C1	C2	1.368(19)
Pb2	I2	3.3996(12)	C4	C3	1.41(2)
Pb2	I2	3.3996(12)	C4	C6	1.393(18)
Pb2	I2	3.1976(8)	C3	C2	1.33(2)
Pb2	I2	3.1976(8)	C6	C6	1.388(18)
Pb2	I1	3.1305(12)			

Table S6. Selected Bond Angles for [1,10-phen][Pb_2I_4].

Atom	Atom	Atom	Angle/°	Atom	Atom	Atom	Angle/°
I2	Pb1	I2	96.26(4)	I1	Pb2	I2	90.72(3)
I1	Pb1	I2	90.21(3)	I1	Pb2	I2	93.69(2)
I1	Pb1	I2	93.55(3)	I1	Pb2	I2	178.88(3)
I1	Pb1	I2	93.55(2)	I1	Pb2	I2	87.94(2)
I1	Pb1	I2	90.21(3)	I1	Pb2	I2	178.87(3)
I1	Pb1	I1	174.37(4)	I1	Pb2	I1	90.94(4)
N1	Pb1	I2	98.7(2)	Pb1	I2	Pb2	85.28(3)
N1	Pb1	I2	164.9(2)	Pb2	I2	Pb1	86.39(2)
N1	Pb1	I2	164.9(2)	Pb2	I2	Pb2	90.69(3)
N1	Pb1	I2	98.7(2)	Pb2	I1	Pb1	89.59(3)
N1	Pb1	I1	86.3(2)	C5	N1	Pb1	118.1(7)
N1	Pb1	I1	89.0(2)	C1	N1	Pb1	125.6(8)
N1	Pb1	I1	89.0(2)	C1	N1	C5	116.3(10)
N1	Pb1	I1	86.3(2)	N1	C5	C5	118.6(6)
N1	Pb1	N1	66.6(4)	N1	C5	C4	121.5(11)
I2	Pb2	I2	89.31(3)	C4	C5	C5	119.9(8)
I2	Pb2	I2	89.31(3)	N1	C1	C2	127.3(13)
I2	Pb2	I2	93.19(3)	C3	C4	C5	116.7(14)
I2	Pb2	I2	86.36(2)	C6	C4	C5	118.9(14)
I2	Pb2	I2	173.71(4)	C6	C4	C3	124.4(13)

I2	Pb2	I2	86.36(2)	C2	C3	C4	120.9(13)
I1	Pb2	I2	87.94(2)	C3	C2	C1	117.2(14)
I1	Pb2	I2	93.69(2)	C6	C6	C4	121.2(7)
I1	Pb2	I2	90.72(3)				

Table S7. Selected Interatomic Distances (Å) for [1,10-phen]$_2$[Pb_5Br_{10}].

Atom	Atom	Length/Å	Atom	Atom	Length/Å
Pb1	Br2	3.1976(12)	N1	C5	1.351(14)
Pb1	Br1	2.8956(13)	N1	C1	1.315(15)
Pb1	N1	2.546(8)	N2	C6	1.367(14)
Pb1	N2	2.522(9)	N2	C7	1.332(15)
Pb2	Br2	3.1813(13)	C5	C6	1.436(15)
Pb2	Br2	2.9271(12)	C5	C4	1.390(15)
Pb2	Br4	3.0511(12)	C6	C10	1.400(15)
Pb2	Br3	3.0760(12)	C1	C2	1.374(17)
Pb2	Br5	2.8912(14)	C7	C8	1.387(18)
Pb2	Br5	3.0813(13)	C4	C3	1.403(17)
Pb3	Br4	3.0150(11)	C4	C12	1.438(16)
Pb3	Br4	3.0150(11)	C10	C11	1.446(16)
Pb3	Br3	2.9943(12)	C10	C9	1.428(17)
Pb3	Br3	2.9943(12)	C3	C2	1.362(17)
Pb3	Br1	3.0385(13)	C11	C12	1.322(18)
Pb3	Br1	3.0386(13)	C9	C8	1.350(19)

12.

Table S8. Selected Bond Angles for [1,10-phen]$_2$[Pb_5Br_{10}].

Atom	Atom	Atom	Angle/°	Atom	Atom	Atom	Angle/°
Br1	Pb1	Br2	77.00(3)	Br1	Pb3	Br1	180.00(4)
N1	Pb1	Br2	132.4(2)	Pb2	Br2	Pb1	113.03(4)
N1	Pb1	Br1	81.9(2)	Pb2	Br2	Pb1	106.61(4)
N2	Pb1	Br2	78.3(2)	Pb2	Br2	Pb2	88.93(3)
N2	Pb1	Br1	103.5(2)	Pb3	Br4	Pb2	100.18(3)
N2	Pb1	N1	65.9(3)	Pb3	Br3	Pb2	135.44(4)
Br2	Pb2	Br2	89.95(3)	Pb1	Br1	Pb3	92.83(4)
Br2	Pb2	Br4	86.60(3)	Pb2	Br5	Pb2	91.57(4)
Br2	Pb2	Br3	79.71(3)	C5	N1	Pb1	118.0(7)
Br2	Pb2	Br5	175.04(4)	C1	N1	Pb1	123.3(8)
Br4	Pb2	Br2	116.41(3)	C1	N1	C5	118.7(9)
Br4	Pb2	Br3	163.67(4)	C6	N2	Pb1	118.0(7)
Br4	Pb2	Br5	96.73(4)	C7	N2	Pb1	122.7(8)
Br3	Pb2	Br2	72.76(3)	C7	N2	C6	119.3(10)
Br3	Pb2	Br5	97.54(4)	N1	C5	C6	118.8(10)
Br5	Pb2	Br2	157.74(4)	N1	C5	C4	121.8(10)
Br5	Pb2	Br2	93.64(4)	C4	C5	C6	119.4(10)
Br5	Pb2	Br2	85.28(3)	N2	C6	C5	119.3(10)
Br5	Pb2	Br4	85.75(4)	N2	C6	C10	121.5(10)
Br5	Pb2	Br3	86.28(4)	C10	C6	C5	119.2(10)
Br5	Pb2	Br5	90.27(4)	N1	C1	C2	123.4(11)
Br4	Pb3	Br4	180.0	N2	C7	C8	122.1(12)
Br4	Pb3	Br1	88.32(3)	C5	C4	C3	117.8(10)
Br4	Pb3	Br1	88.32(3)	C5	C4	C12	119.7(11)
Br4	Pb3	Br1	91.68(3)	C3	C4	C12	122.5(11)
Br4	Pb3	Br1	91.68(3)	C6	C10	C11	119.8(10)
Br3	Pb3	Br4	100.11(3)	C6	C10	C9	117.5(11)
Br3	Pb3	Br4	79.89(3)	C9	C10	C11	122.6(11)
Br3	Pb3	Br4	100.11(3)	C2	C3	C4	119.5(11)
Br3	Pb3	Br4	79.89(3)	C3	C2	C1	118.9(11)
Br3	Pb3	Br3	180.0	C12	C11	C10	120.2(11)
Br3	Pb3	Br1	96.40(3)	C8	C9	C10	119.4(12)
Br3	Pb3	Br1	96.40(3)	C9	C8	C7	120.3(13)
Br3	Pb3	Br1	83.60(3)	C11	C12	C4	121.5(11)
Br3	Pb3	Br1	83.60(3)				

Table S9. Selected Interatomic Distances (Å) for [1,10-phenH][SbI_4]·(H_2O).

Atom	Atom	Length/Å	Atom	Atom	Length/Å
Sb1	I1	2.8287(4)	C8A	C12A	1.377(4)
Sb1	I2	2.8745(4)	C9A	C10A	1.363(5)
Sb1	I3	3.2627(4)	C10A	C11A	1.379(5)
Sb1	I3	3.0255(4)	N1B	C1B	1.384(4)
Sb1	I4	3.0331(4)	N1B	C2B	1.382(4)
Sb1	I4	3.2675(4)	N2B	C11B	1.389(4)
N1A	C1A	1.371(4)	N2B	C12B	1.377(4)
N1A	C2A	1.361(4)	C1B	C5B	1.371(4)
N2A	C11A	1.355(4)	C1B	C12B	1.439(4)
N2A	C12A	1.368(4)	C2B	C3B	1.376(4)
C1A	C5A	1.384(4)	C3B	C4B	1.381(5)
C1A	C12A	1.439(4)	C4B	C5B	1.361(4)
C2A	C3A	1.381(5)	C5B	C6B	1.457(6)
C3A	C4A	1.376(4)	C6B	C7B	1.358(9)
C4A	C5A	1.382(4)	C7B	C8B	1.447(6)
C5A	C6A	1.461(4)	C8B	C9B	1.355(4)
C6A	C7A	1.343(6)	C8B	C12B	1.368(4)
C7A	C8A	1.441(5)	C9B	C10B	1.379(5)
C8A	C9A	1.389(4)	C10B	C11B	1.363(5)

Table S10. Selected Bond Angles for [1,10-phenH][SbI_4]·(H_2O).

Atom	Atom	Atom	Angle/°	Atom	Atom	Atom	Angle/°
I1	Sb1	I2	95.297(9)	C12A	C8A	C7A	121.2(3)
I1	Sb1	I3	91.316(9)	C12A	C8A	C9A	119.2(3)
I1	Sb1	I3	93.552(9)	C10A	C9A	C8A	120.4(3)
I1	Sb1	I4	172.423(9)	C9A	C10A	C11A	119.5(3)
I1	Sb1	I4	88.006(9)	N2A	C11A	C10A	120.3(3)
I2	Sb1	I3	89.661(11)	N2A	C12A	C1A	120.1(3)
I2	Sb1	I3	171.181(8)	N2A	C12A	C8A	119.8(2)
I2	Sb1	I4	94.442(11)	C8A	C12A	C1A	120.1(2)
I2	Sb1	I4	90.370(9)	C2B	N1B	C1B	117.5(3)
I3	Sb1	I3	84.100(10)	C12B	N2B	C11B	119.2(3)
I3	Sb1	I4	83.587(8)	N1B	C1B	C12B	118.5(2)
I3	Sb1	I4	175.462(8)	C5B	C1B	N1B	123.2(2)
I3	Sb1	I4	91.502(9)	C5B	C1B	C12B	118.2(2)
I4	Sb1	I3	91.608(10)	C3B	C2B	N1B	120.6(3)
I4	Sb1	I4	86.542(9)	C2B	C3B	C4B	119.3(3)
Sb1	I3	Sb1	95.899(11)	C5B	C4B	C3B	121.9(3)
Sb1	I4	Sb1	93.458(9)	C1B	C5B	C6B	122.0(4)
C2A	N1A	C1A	117.4(3)	C4B	C5B	C1B	117.4(3)
C11A	N2A	C12A	120.8(3)	C4B	C5B	C6B	120.6(4)
N1A	C1A	C5A	123.2(2)	C7B	C6B	C5B	119.0(7)
N1A	C1A	C12A	118.2(2)	C6B	C7B	C8B	119.2(7)
C5A	C1A	C12A	118.5(2)	C9B	C8B	C7B	117.8(4)
N1A	C2A	C3A	121.9(3)	C9B	C8B	C12B	120.8(3)
C4A	C3A	C2A	119.3(3)	C12B	C8B	C7B	121.3(4)
C3A	C4A	C5A	120.6(3)	C8B	C9B	C10B	120.3(3)
C1A	C5A	C6A	120.2(3)	C11B	C10B	C9B	119.5(3)
C4A	C5A	C1A	117.5(3)	C10B	C11B	N2B	120.4(3)
C4A	C5A	C6A	122.3(3)	N2B	C12B	C1B	120.1(2)
C7A	C6A	C5A	120.8(4)	C8B	C12B	N2B	119.8(2)
C6A	C7A	C8A	119.0(4)	C8B	C12B	C1B	120.1(3)
C9A	C8A	C7A	119.6(3)				
